# Integrated structural, optical and dielectric analysis of low-loss α-Al₂O₃ nanoparticles for UV photonic and dielectric applications

**DOI:** 10.1038/s41598-026-50503-4

**Published:** 2026-05-11

**Authors:** Sara A. Mohamed, Abdalrahman M. Rayan, A M Abdel Hakeem, S. H. Mohamed, A. M. Abd El-Rahman, Khater A. E. Gad, Mahrous R. Ahmed, Naglaa AbdelAll

**Affiliations:** 1 Physics Department, Faculty of Science, Sohag University, Sohag, 82524 Egypt; 2 Department of Mathematical Statistics, Faculty of Graduate Studies for Statistical Research, Cairo University, Giza, Egypt; 3 Physics Department, Faculty of Science, Assiut University, Assiut, 71516 Egypt

**Keywords:** α-Al_2_O_3_ nanoparticles, Optical band gap, Dielectric loss, Complex dielectric function, Extinction coefficient, Low-loss dielectrics, Sol–gel synthesis, Materials science, Nanoscience and technology, Optics and photonics, Physics

## Abstract

High-purity α-Al2O3 nanoparticles were synthesised using a modified Pechini sol–gel method and calcinated at 1100 °C. Their structural, optical, and dielectric properties were thoroughly examined. Structure research utilizing X-ray diffraction and advanced Rietveld refinement showed that adding the axial divergence asymmetry pseudo-Voigt function improves refinement quality and produces more precise crystallographic parameters for the R-3c corundum structure. A stress-free crystalline framework was confirmed by a size-strain plot showing a volume-weighted crystallite size of ~ 24.4 nm and low lattice strain. The HR-TEM revealed spherical polycrystalline aggregates with an average particle size of ~ 100 nm, while FTIR confirmed phase purity and complete organic precursor elimination. UV–Vis diffuse reflectance spectroscopy determined the refractive index, extinction coefficient, absorption coefficient, and complex dielectric function. The Kubelka–Munk formalism estimated the optical band gap at ~ 4.29 eV, indicating wide-band-gap insulating behaviour. In the visible-near-infrared region, the real part of the dielectric constant showed substantial photon energy dispersion, but the imaginary part remained extremely low (≤ 0.03), indicating minimal optical losses. The dielectric loss tangent was extremely low (~ 10–4–10–6), indicating strong electronic polarization and minimal dissipation. The lattice dielectric constant (εₗ = 7.97), low plasma frequency, and minimal free-carrier contribution support the intrinsic insulation of α-Al2O3. Researchers found a strong correlation between structural perfection and low-loss optical response, making α-Al2O3 nanoparticles promising for high-transparency, dielectric-stable applications such as optical coatings, ultraviolet optoelectronic devices, and high-frequency photonics.

## Introduction

Alpha alumina oxide (α- Al_2_O_3_), commonly known as alpha alumina or corundum, is the most thermodynamically stable phase of alumina oxide^1^. Among its exceptional physical and chemical properties are high hardness, exceptional thermal stability, chemical inertness, and excellent electrical insulation^2^. Because of these properties, α- Al_2_O_3_ is essential for many industrial and technological applications, including reinforcement^3^, catalysts^4^, refractories^5^, and adsorbents^6^, the fabrication of reinforcements, electronics, photonics, sensors, and catalysts^7^. In the α-AlO₃ hexagonal close-packed crystal structure, alumina cations occupy particular interstitial locations among oxygen anions^8^. This well-organized and compact crystal structure provides remarkable stability in comparison to other metastable alumina phases such as γ-Al₂O₃ and δ- Al_2_O_3_^9^. Because of this, α- Al_2_O_3_ is the common phase of alumina and is being studied in great detail for both fundamental and practical uses. α- Al_2_O_3_ can be prepared in several methods, such as hydrothermal-assisted freeze-drying^10^, isopropanol through thermal treatment^11^. ultrasonic spray pyrolysis^11^ Bayer method^12^, precipitation method^13^. Heating the transitional alumina phases (γ, δ, θ) causes them to gradually transform into the stable α-phase^14^. precipitation of inorganic aluminum salts^15^. Sol–Gel Method^16–19^.

The Pechini method, also known as the polymerizable complex route, is a versatile and effective chemical synthesis strategy for the production of high-purity, nanocrystalline metal oxides. This technique is based on the formation of a stable metal-chelate complex between metallic cations and a chelating agent, typically a hydroxycarboxylic acid like citric acid^20,21^. The process proceeds through a polyesterification reaction between the metal-citrate complexes and a polyhydroxy alcohol, such as ethylene glycol or polyethylene glycol (PEG). This reaction results in a rigid, three-dimensional polymeric resin that uniformly traps the metal ions within its network. The primary advantage of this immobilization is the prevention of metal ion segregation at the molecular level, which ensures high chemical homogeneity throughout the precursor. Upon subsequent thermal treatment at high temperatures, the organic matrix undergoes oxidative decomposition, facilitating the phase transformation into the thermodynamically stable α-Alumina (corundum) structure. This method is particularly favoured for synthesizing alumina nanoparticles because it allows for the development of a well-crystallized lattice with controlled particle morphology and minimized structural defects^22,23^.

Rietveld refinement is an indispensable analytical technique in modern crystallography that allows for the complete structural characterization of polycrystalline materials by minimizing the difference between an observed diffraction pattern and an idealized calculated model^24–28^. Unlike traditional peak-matching methods, the Rietveld approach utilizes the entire diffraction profile, incorporating complex parameters such as lattice constants, atomic fractional coordinates, and site occupancy factors to provide a holistic view of the unit cell.

In the refinement of polycrystalline diffraction data, the selection of an appropriate profile fitting function is paramount, as it dictates the accuracy of the extracted structural and microstructural information. The Pseudo-Voigt (p_V_) function is widely favoured in Rietveld analysis due to its ability to approximate the complex diffraction line shapes by linearly combining Gaussian and Lorentzian components, which respectively account for different sources of peak broadening^29,30^.

However, standard symmetric functions often fail to accurately model the peak profiles at low 2θ angles, where instrumental geometry induces significant axial divergence asymmetry. To overcome this, the use of a modified profile function that incorporates an asymmetry correction is essential. This approach allows for the mathematical deconvolution of instrumental aberrations such as those caused by finite slit widths and beam divergence from the physical broadening inherent to the sample. By utilizing an asymmetric fitting model, the refinement process can more precisely locate peak positions and intensities, leading to more reliable determinations of lattice parameters, atomic positions, and microstructural properties like crystallite size and lattice strain. Ultimately, the transition from a symmetric to an asymmetric profile function ensures that the final structural model is a truer representation of the physical specimen rather than a result of instrumental artifacts^31,32^.

Precise structural refinement is fundamental to the development of low-loss dielectric materials because the energy dissipation mechanisms in these systems are intrinsically linked to atomic-scale defects and symmetry deviations. In α-Al₂O₃, dielectric loss is often driven by extrinsic factors such as oxygen vacancies, lattice strain, or local structural distortions that create dipoles capable of oscillating under an applied electromagnetic field^33–35^. Standard diffraction models often fail to capture these nuances, leading to an incomplete understanding of the material’s polarization behavior. By employing advanced Rietveld refinement specifically utilizing axial divergence asymmetry and accurate peak-shape modeling, researchers can precisely determine lattice parameters, atomic positions, and isotropic displacement factors. This level of detail allows for a rigorous evaluation of the crystalline framework’s integrity, ensuring that the synthesized nanoparticles possess the high phase purity and stress-free structure (as evidenced by negligible lattice strain) required to minimize vibrational damping and electronic leakage. Consequently, such refinement serves as a predictive tool for achieving the ultra-low loss tangents (tanδ ≈10^–4^–10^–6^) necessary for high-frequency and high-power applications^36,37^.

In high-power electronics and UV photonics, α-Al₂O₃ (corundum) serves as a critical wide-bandgap material due to its exceptional thermal stability and optical transparency. For high-power electronic applications, its high dielectric strength and superior thermal conductivity are essential for efficient heat dissipation and electrical insulation in power MOSFETs and gate dielectrics^38,39^, where it helps mitigate leakage currents under extreme operating conditions. Simultaneously, in the field of UV photonics, the wide optical bandgap of α-Al₂O₃ (approximately 4.29 eV in this study) allows for high transmission in the deep-ultraviolet spectrum, making it an ideal substrate or protective coating for UV light-emitting diodes (LEDs) and solar-blind photodetectors^40,41^. The integration of high-purity α-Al₂O₃ nanoparticles into these technologies is particularly advantageous, as their low dielectric loss and stress-free crystalline framework enhance the overall efficiency and longevity of devices operating in harsh, high-energy environments.

The primary objective of this study is to provide a rigorous structural and microstructural investigation of high-purity α-Alumina nanoparticles synthesized via a modified Pechini sol–gel route. By employing advanced Rietveld refinement techniques, this work aims to evaluate the efficacy of the axial divergence asymmetry profile fitting function in resolving instrumental aberrations from intrinsic specimen broadening. Furthermore, the study seeks to correlate the refined lattice parameters and internal polyhedral geometries (AlO_6_ octahedra) with the vibrational fingerprint obtained through FTIR spectroscopy in ATR mode. Through a systematic Size-Strain Plot (SSP) analysis, we intend to quantify the crystallite size and investigate the presence of lattice strain following 1100 °C calcination, thereby establishing a comprehensive understanding of the phase purity and structural evolution of corundum at the nanoscale.

This study introduces a comprehensive structure–optical–dielectric correlation in α- Al_2_O_3_ nanoparticles, emphasizing the intrinsic origins of optical transparency and dielectric loss suppression. Unlike previous reports that focus on isolated optical constants, the present work simultaneously evaluates the refractive index, extinction coefficient, dielectric function, and loss tangent within a unified framework. Improved crystallographic refinement using axial divergence asymmetry provides a robust structural basis for interpreting optical dispersion and loss behavior. The resulting low extinction coefficient, minimal dielectric loss, and wide optical band gap demonstrate that α- Al_2_O_3_ is intrinsically optimized for ultraviolet optoelectronics, photonic structures, and dielectric applications requiring high transparency and long-term stability.

## Results and discussion

### Material synthesis

The α-phase aluminium oxide (α-Al_2_O_3_) nanoparticles were synthesized via a modified Pechini sol–gel method. In a typical procedure, aluminium nitrate Nona hydrate [Al(NO_3_)_3_.9H_2_O] was employed as the metallic precursor, with citric acid and polyethylene glycol (PEG 400) serving as the chelating agent and polymerization agent, respectively. All chemicals used were purchased from the Pio-Chem company, respectively. The precursors were dissolved in deionized water under vigorous stirring. To facilitate polyesterification, the solution pH was adjusted to 9.0 by the dropwise addition of an aqueous ammonia solution. The resulting mixture was subjected to continuous magnetic stirring at 850 RPM for 5 h, maintained at a constant temperature of 90°C to promote the formation of a homogeneous polymeric resin. Subsequently, the solution was dehydrated at 200°C for 12 h until a voluminous black ash (xerogel) was obtained. This intermediate product was ground into a fine powder and subjected to calcination at 1100°C for 8 h in an atmospheric furnace to ensure complete organic burnout and the phase transformation to the thermodynamic α-corundum phase. The final product was obtained as a high-purity white powder.

### Characterization

**Fig. 1:**
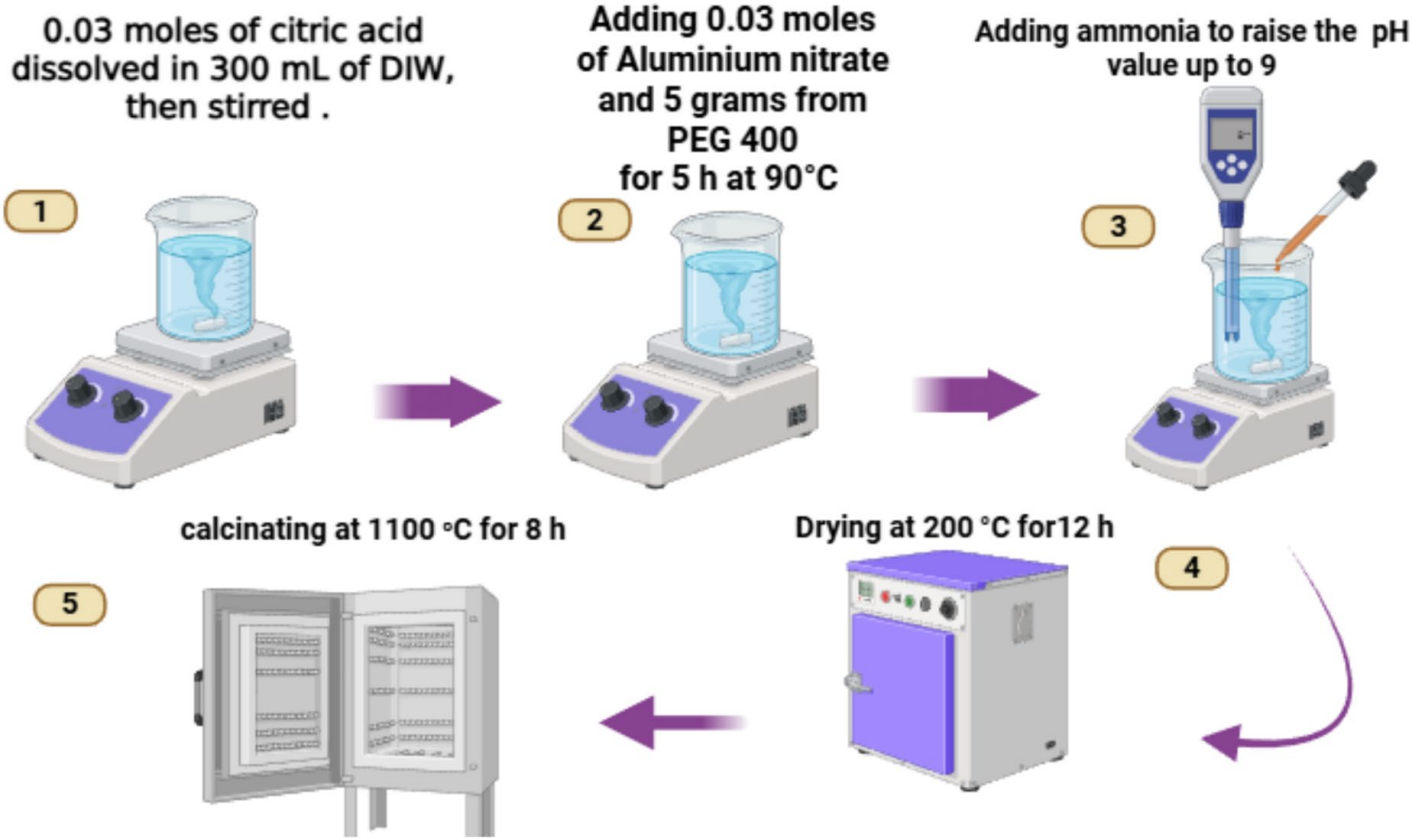
The reaction diagram for α-alumina synthesis.

The crystalline structure and phase purity of the synthesized powder were evaluated via X-ray diffraction (XRD). Morphological features and lattice fringes were analysed using High-Resolution Transmission Electron Microscopy (HR-TEM). Chemical bonding and functional groups were identified through Fourier-transform infrared spectroscopy (FTIR). Furthermore, the optical properties and bandgap characteristics were investigated using UV–Vis-IR diffuse reflectance spectroscopy. Figure 1 illustrates the reaction diagram for α-Alumina synthesis.

### Reaction diagram

### Phase analysis

**Fig. 2:**
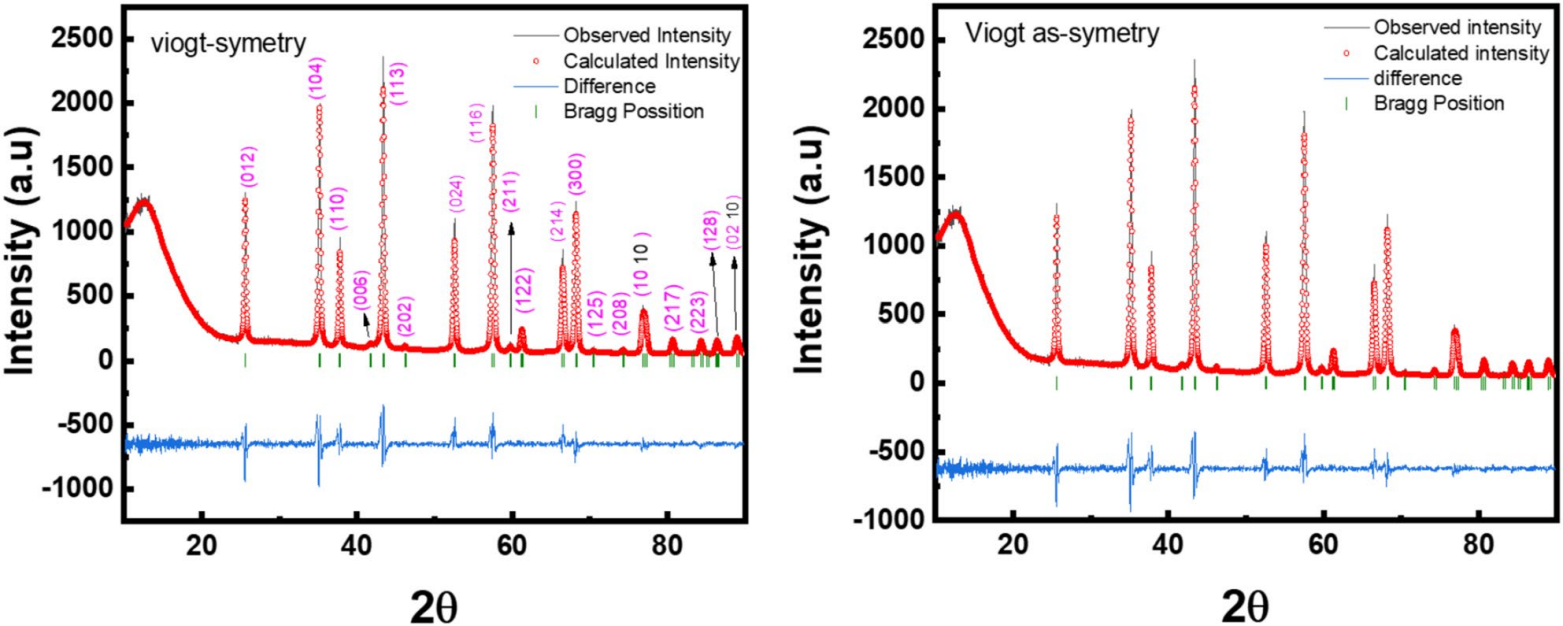
The rietveld refinement patterns for the α-alumina sample using the pseudo-Voigt and pseudo-Voigt axial divergence asymmetry.

The crystalline structure of the synthesized α-Alumina nanoparticles was characterized by X-ray diffraction (XRD) using the Bragg–Brentano geometry. Data collection was performed over a 2θ range from 10° to 90° with a precise step size of 0.02°. Initial phase identification and pattern matching were executed using X’pert High Score Plus software, which confirmed that the diffraction peaks perfectly coincide with the rhombohedral corundum phase, indexed under ICSD collection code: 030,024. Subsequently, a comparative whole-pattern fitting was conducted via Rietveld refinement in Fullprof to evaluate the profile modelling. To further investigate the microstructural properties, a comparative whole-pattern fitting was executed through Rietveld refinement, specifically evaluating two different profile functions, pseudo-Voigt and pseudo-Voigt axial divergence asymmetry. Figure 2 illustrates the refinement for the α-Alumina sample using the two functions.

The diffraction data were collected from 10° to 90° with a precise step size of 0.02°. showed high crystallinity. A comparison of the refinement results indicates that the Voigt asymmetry function provided a superior fit compared to the symmetric model. Specifically, the goodness of fit (χ^2^) decreased from 2.6 to 2.48, and the weighted profile R-factor (R_wp_) improved from 21.9% to 21.5%. Visually, the symmetric Voigt fit exhibited significant S-shaped residuals in the difference plot at the base of the major reflections, such as the (104) and (113) planes, revealing a failure to model the peak skewness. The application of the asymmetric function effectively suppressed these residuals, better accommodating the instrumental axial divergence and geometric broadening characteristic of the Bragg–Brentano setup.

Furthermore, the refined lattice parameters (a = b approximately 4.762 ± 0.001 Å and c approximately 13.002 ± 0.001 Å) and unit cell volumes remained consistent across both models, confirming the high phase stability and purity of the corundum product. The comparative refinement revealed a notable shift in the calculated theoretical density, moving from 4.115 g/cm^3^ in the symmetric model to 3.985 g/cm^3^ in the asymmetric model. While the unit cell volume remained stable at approximately 255.3 Å^3^, the improved reliability factors (χ^2^ = 2.48) in the asymmetric fit suggest that this model better captures the real-world state of the nanoparticles. The lower theoretical density in the asymmetric refinement likely reflects the sensitivity of the model to peak tailing and potential lattice irregularities inherent in α-Al2O3 synthesized via the Pechini method and calcined at 1100 °C. Table 1 summarizes the refined parameters for the fitting functions.

**Table 1:** The refined parameters for the fitting functions.

Pseudo Voigt		Pseudo Voigt “axial divergence asymmetry”	
Crystal system	Rhombohedral	Crystal system	Rhombohedral
Space group	R-3c	Space group	R-3c
Lattice parameters	a = b = 4.762379 ± 0.001 Å c = 13.002892 ± 0.003 Å α = β = 90^o^ γ = 120^o^	Lattice parameters	a = b = 4.7622 ± 0.001 Å c = 13.0021 ± 0.003 Å α = β = 90^o^ γ = 120^o^
Unit cell volume	255.3987 ± 2.035Å^3^	Unit cell volume	255.3675 ± 2.035 Å^3^
Theoretical density	4.115 g/cm^3^	Theoretical density	3.985 g/cm^3^
R-factors	R_p_: 25.5 R_wp_: 21.9 R_exp_: 13.59	R-factors	R_p_: 25.1 R_wp_: 21.5 R_exp_: 13.65
Goodness of fit (χ^2^)	2.6	Goodness of fit (χ^2^)	2.48

To get deeper into the reason we have chosen the two functions for the fitting, we should first know their properties from a mathematical and physical point of view. In Fullprof, the pseudo-Voigt and axial divergence asymmetry functions represent two distinct mathematical approaches for modelling the physical reality of a diffraction peak. The pseudo-Voigt function is primarily used to model the intrinsic broadening of the peak, which is physically caused by the instrument’s inherent resolution and the sample’s microstructural characteristics, such as finite crystallite size and lattice strain^42,43^. Mathematically, the pseudo-Voigt Ω is defined as a linear combination of a Gaussian (G) and a Lorentzian (L) function, both sharing the same Full Width at Half Maximum (FWHM), denoted as H. This function is expressed as^44^:

1 $$\Omega \left( x \right) \, = \, \eta L\left( {x,H} \right) \, + \, \left( {1 - \eta } \right) \, G\left( {x,H} \right)$$

where *η* is the mixing parameter that determines the “shape” of the peak, with *η* = 0 representing a pure Gaussian and *η* = 1 a pure Lorentzian profile. The axial divergence asymmetry addresses a completely different physical phenomenon, the geometric distortion of peaks, which is most prominent at low scattering angles. This asymmetry arises because the X-ray or neutron beam is not a mathematical line but has a vertical height, causing the curved Debye–Scherrer cones to be intercepted by the finite height of the detector slits in a way that “smears” the intensity toward lower angles.

In Fullprof, this is often treated using the Finger-Cox-Becker model^45,46^. Unlike the pseudo-Voigt, which is inherently symmetric around the Bragg angle, the axial divergence function is an asymmetric correction. Mathematically, it is treated as a convolution of the symmetric peak shape (the pseudo-Voigt) with an asymmetry function that depends on the physical dimensions of the diffractometer, such as the sample and slit heights relative to the goniometer radius. From a refinement perspective, these functions are controlled by different parameters in the program’s input control file (.pcr). The pseudo-Voigt width is typically governed by the Caglioti parameters (U, V, W), which model how the Gaussian FWHM (H_G_) changes with the scattering angle θ^47,48^:

2 $${H}_{G}^{2}=U {tan}^{2}\theta +tan\theta +W$$

In contrast, axial divergence asymmetry is corrected using physical geometric parameters (often labelled as H/L and S/L While the pseudo-Voigt broadening generally increases with the scattering angle, the axial divergence asymmetry is most severe at low angles and diminishes as θ increases. By combining these two mathematical forms, Fullprof can accurately simulate the complex shapes of experimental diffraction peaks across the entire recorded range.

The Rietveld refinement was performed depending on the Young Algorithm, which utilizes a derivative of the DBW (Wiles & Young) program code for its core computational routines^49,50^. This algorithm employs a non-linear least squares minimization technique to optimize the agreement between the observed powder diffraction pattern (y_obs_) and a calculated profile (y_calc_) based on a structural model. Specifically, the program minimizes the weighted residual function through a series of iterative cycles, in which the parameter shifts are determined by solving the normal equations. The refinement strategy followed the established protocols proposed by Wiles and Young, prioritizing the convergence of the scale factor, background parameters, and lattice constants before proceeding to refine atomic coordinates, thermal displacement parameters, and profile-specific variables such as peak shape and asymmetry. Herein, the geometrical parameters, including the atomic positions, thermal factors, and Occupancy are extracted from the refinement and illustrated in Tables 2 and 3.

**Table 2:** The geometrical parameters for the Pseudo Voigt fitting.

Parameters	Pseudo Voigt			
Atomic positions		*x*	*y*	*z*
	Al	0.0	0.0	0.35248
	O	0.31034	0.0	0.25
Thermal factor (isotropic)	Al		0.05119	
	O		0.17409	
Occupancy	Al		0.33074	
	O		0.53239	
Zero- shift	-0.072740			
FWHM parameters	U	V	W	η
	0.132065	0.037658	0.056656	0.000061
Shape parameters	Eta_0_		X	
	0.717690		-0.003913	
Scale factor	0.11592 x 10^-2^

**Table 3:** The geometrical parameters for the Pseudo Voigt “axial divergence asymmetry” fitting.

parameters	Pseudo Voigt “axial divergence asymmetry.”							
Atomic positions		*x*	*y*			*z*		
	Al	0.0	0.0			0.35273		
	O	0.31023	0.0			0.25		
Thermal factor (isotropic)	Al		B_11_	B_22_	B_33_	B_12_	B_13_	B_23_
			0.0500	0.0500	0.0312	0.00502	0.0	0.0
	O		B_11_	B_22_	B_33_	B_12_	B_13_	B_23_
			0.00481	0.0	0.00424	0.0	0.061	0.0569
Occupancy	Al		0.30362					
	O		0.50366					
Zero- shift	-0.074160							
FWHM parameters	U	V	W			η		
	0.121527	0.054651	0.081790			0.023062		
Shape parameters	Eta_0_				X			
	0.676690				-0.003022			
Scale factor	0.14176 x 10^-2^							

The Rietveld refinement results for rhombohedral Al_2_O_3_ (Space Group R-3c) demonstrate a significant dependence on the chosen profile function, specifically regarding the treatment of peak asymmetry. The transition from a standard Pseudo-Voigt function to a Pseudo-Voigt incorporating axial divergence asymmetry (Finger-Cox-Jephcoat correction) reflects a more physically accurate representation of the diffractometer’s optical geometry. In the standard model, the vertical spread of the X-ray beam prevalent at lower 2θ angles is not explicitly modelled, often leading to a systematic bias in the Caglioti FWHM parameters (U, V, W) and the Lorentzian mixing factor (η) as the algorithm attempts to fit asymmetric experimental profiles with symmetric mathematical functions. Refinement using the axial divergence model resulted in a redistribution of intensity that altered the structural and global parameters.

Notably, the scale factor increased from 0.11592 × 10^–2^ to 0.14176 × 10^–2^, while the calculated atomic occupancies decreased (e.g., Al occupancy shifted from 0.33074 to 0.30362). This shift, combined with the transition from isotropic (B_iso_) to anisotropic thermal factors (B_ij_), allows for a higher degree of freedom in describing atomic displacement. However, the emergence of negative values for certain anisotropic components (e.g., B_11_ = -0.01106 for Al) indicates a non-physical refinement state, likely over-compensating for underlying experimental factors such as absorption or background noise. From a statistical standpoint, the axial divergence model provided a superior fit to the experimental data. As shown in Table 1, the Goodness of Fit (χ^2^) improved from 2.6 to 2.48, accompanied by a decrease in the weighted profile R-factor (R_wp_) from 21.9 to 21.5. While the lattice parameters and unit cell volume remained relatively stable, the theoretical density exhibited a marked decrease from 4.115 g/cm^3^ to 3.985 g/cm^3^. This suggests that while the asymmetric model is mathematically more robust and offers better convergence, the resulting physical parameters, specifically density and anisotropic vibrations, must be interpreted with caution relative to the standard isotropic model.

**Fig. 3:**
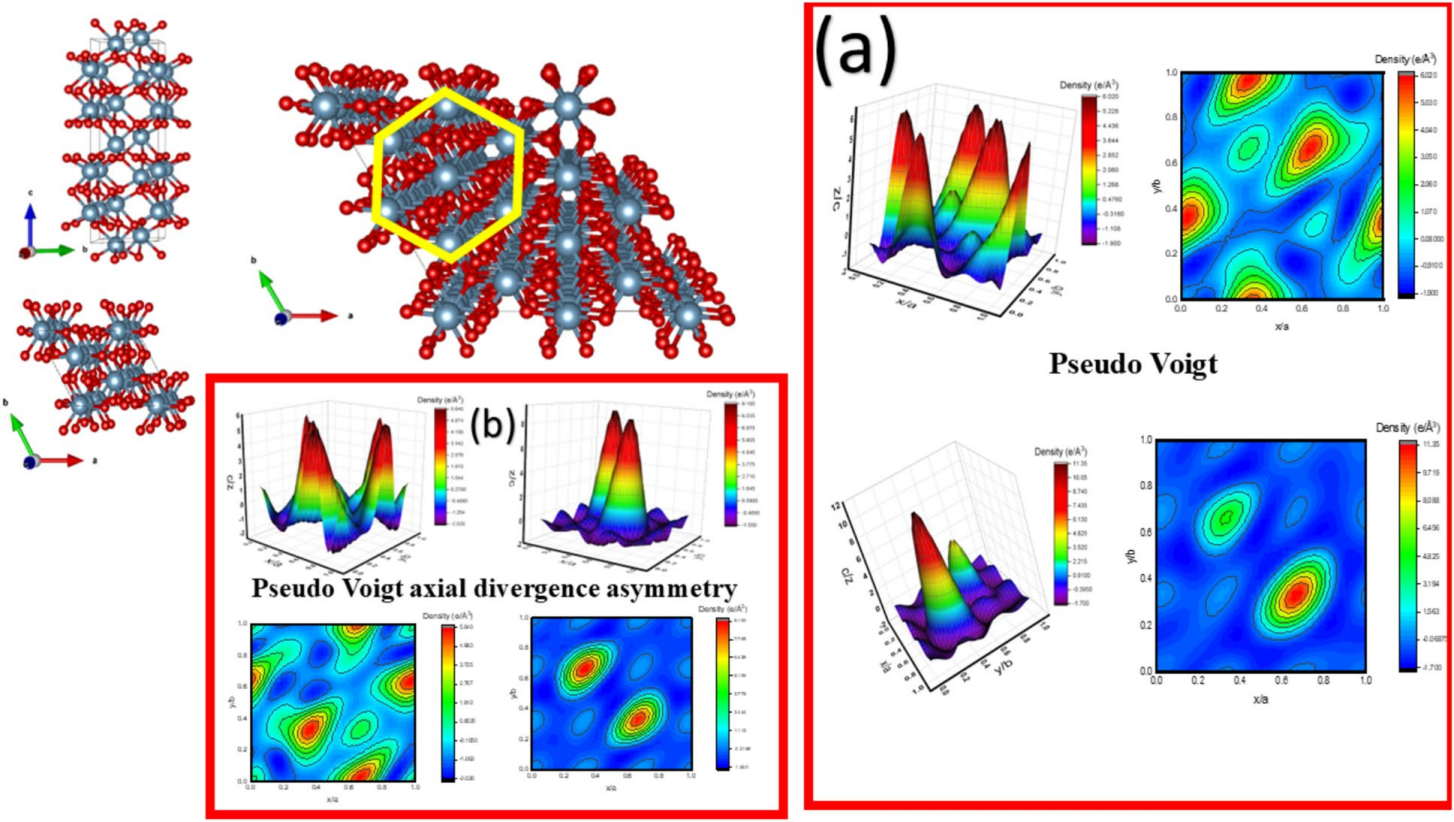
(**a**) the EDM for the pseudo-Viogt fitting function; (**b**) the EDM for the pseudo-Viogt axial divergence asymmetry fitting function.

The electron density mapping (EDM) visually confirms the mathematical superiority of the axial divergence asymmetry function. According to the following equation, the EDM can be visualized^51,52^.

3 $$\uprho \left(\mathrm{r}\right)=\frac{1}{V} \sum_{H}F\left(H\right).{e}^{-2\pi i(H.r)}$$

where V is the unit cell volume, r is the position vector, H is the reciprocal lattice vector, and F(H) is the complex Fourier function. In the standard Pseudo-Voigt maps (Fig. 3,a), the electron density peaks appear broadened and are surrounded by significant Fourier artifacts or “noise” ripples in the interstitial regions. Conversely, the maps generated using the axial divergence asymmetry function (Fig. 3, b) exhibit significantly sharper, more localized density maxima and a much smoother background. This clarity is attributed to the more accurate modelling of the anisotropic thermal factors (B_ij_) as detailed in Table 3, which allows for a more realistic representation of the atomic electron distribution compared to the isotropic constraints used in the standard model. The primary reason for the enhanced performance of the Pseudo-Voigt "axial divergence asymmetry" function lies in its ability to account for the geometric constraints of the diffractometer.

Standard Pseudo-Voigt functions assume a symmetric peak shape; however, in real X-ray diffraction experiments, peaks, especially at low 2θ angles, experience broadening and tailing due to the vertical (axial) divergence of the X-ray beam. By incorporating an asymmetry correction, the refinement process mathematically compensates for this instrumental "smearing." This prevents the intensity belonging to the peak tails from being misinterpreted as background or displaced density, thereby leading to a more accurate Fourier transformation during the generation of electron density maps. Consequently, the axial divergence model provides a truer physical representation of the α-Alumina crystal structure by correctly localizing the electron density at the atomic sites and minimizing artificial electronic blurring.

**Fig. 4:**
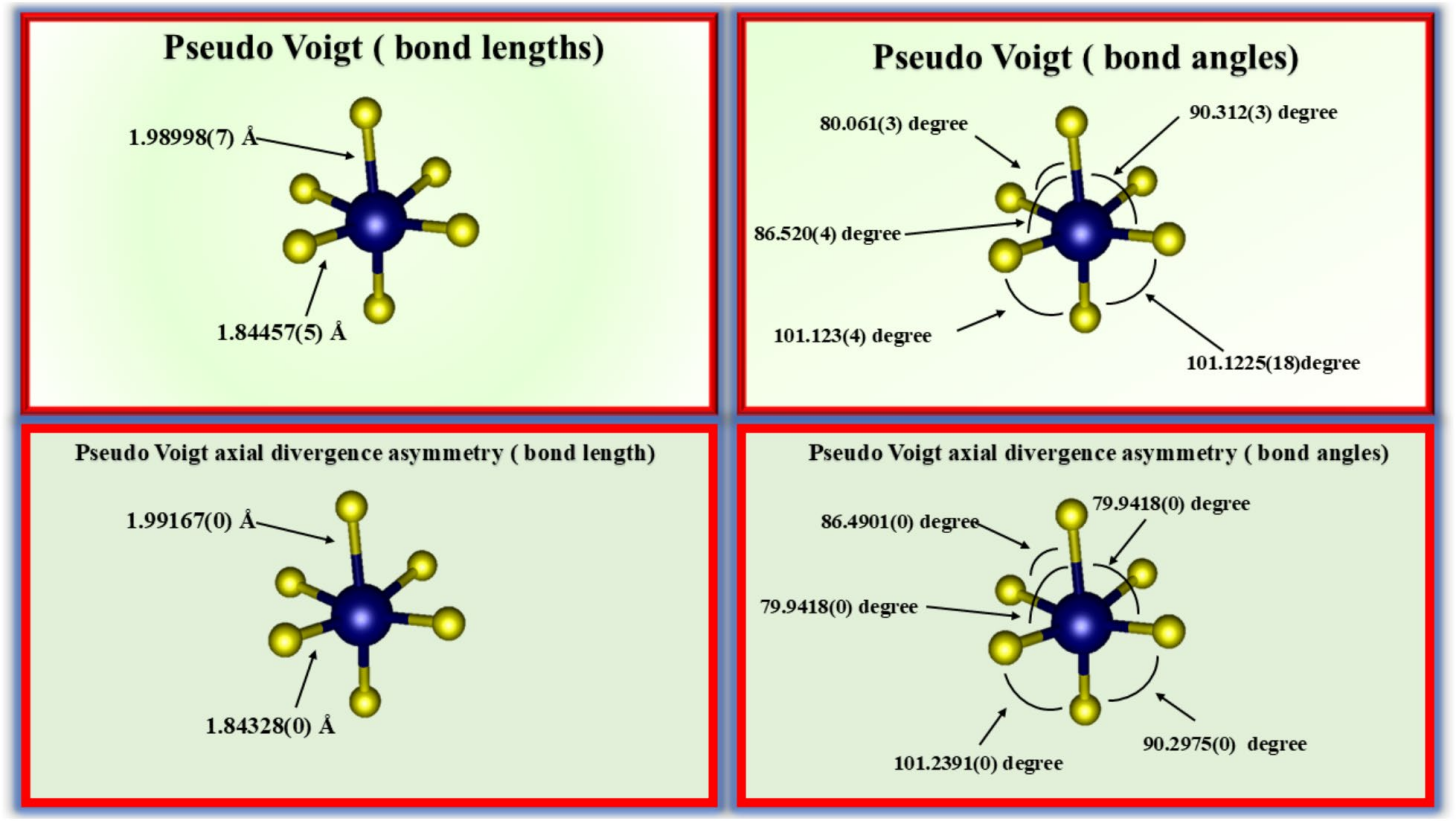
The bond angles and lengths for the two fitting functions.

The local coordination environment of the Al^3+^ cations and O^2-^ anions in the rhombohedral α-Alumina structure was further examined by calculating the Al-O bond lengths and O–Al-O bond angles derived from both fitting functions. In the corundum structure (R-3c), each aluminum atom is octahedrally coordinated by six oxygen atoms, forming two distinct sets of Al–O bond lengths due to the trigonal distortion^53^. Figure 4 illustrates the bond angles and lengths for the two fitting functions.

### Microstructural study

According to Bragg’s law for diffraction, the D- spacing is governed by the peak position as follows^54^;

4 $${\mathrm{n}}\lambda \, = {\text{ 2dsin }}\theta$$

where n is the order of diffraction, λ is the intrinsic wavelength of the K_α1_ of the diffractometer, in our case, the target is copper, and the value of wavelength is taken as 1.5406 Å, d is the interplanar spacing between the atomic planes measured in angstroms, and θ is the angle of the incident beam in degrees. The type of fitting function plays an important role in determining the peak center and thereby the value of the d-spacing. Going further, the peak center also controls the value of the full width at half maximum (FWHM), which is an important player in the microstructural calculations. The following table contains the values of the estimated values of peak positions, d-spacing, and FWHM for the α-Alumina using the two fitting functions.

**Fig. 5:**
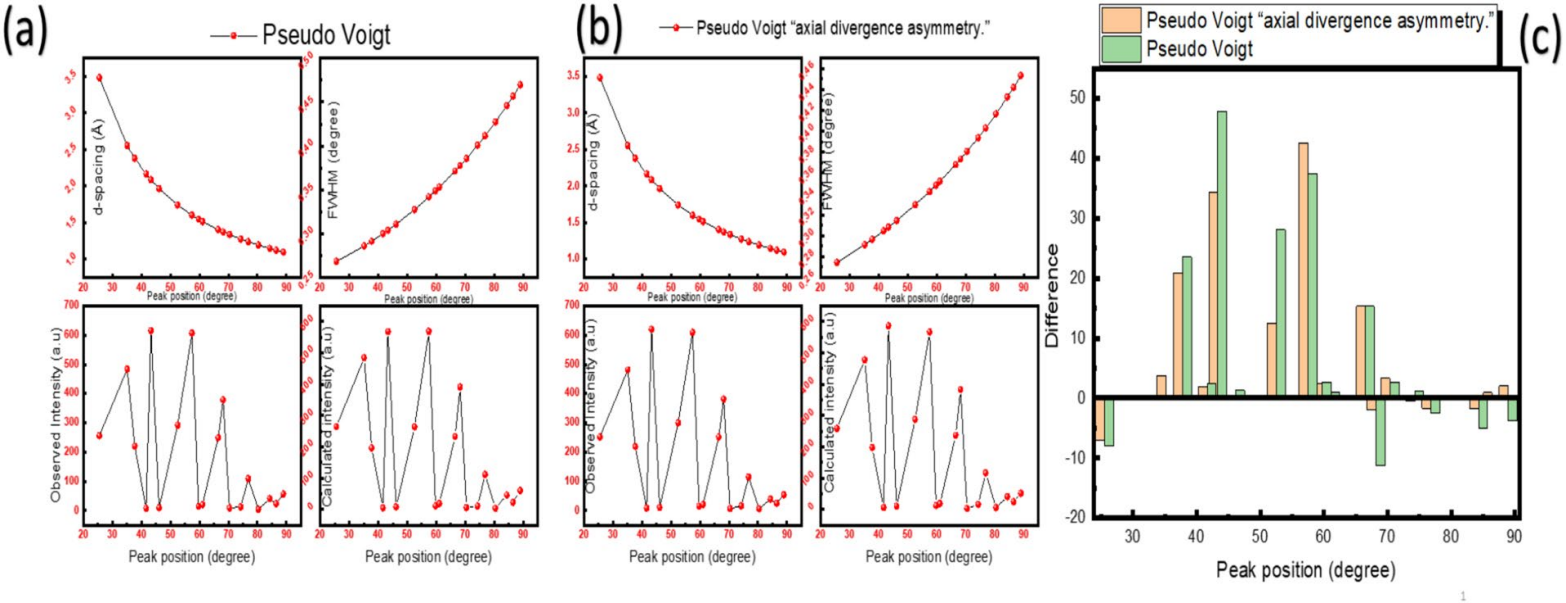
The difference in the parameters in Table 4.

The Rietveld refinement of the synthesized α-Alumina nanoparticles reveals a strong correlation between the chosen profile function and the accuracy of the structural model. While the initial phase identification confirmed a high-purity rhombohedral corundum phase (ICSD: 030,024), the comparative whole-pattern fitting demonstrates that the standard symmetric pseudo-Voigt function is insufficient to model the instrumental effects inherent in the Bragg–Brentano geometry. Specifically, the symmetric model exhibited significant S-shaped residuals at the base of high-intensity reflections such as the (104) and (113) planes, indicating a failure to account for axial divergence. As illustrated in Fig.  2 and supported by the peak-specific data, the application of the axial divergence asymmetry (Finger-Cox-Jephcoat correction) yielded a superior fit. This transition resulted in a quantitative improvement in the reliability factors, with the goodness of fit χ^2^ decreasing from 2.6 to 2.48 and the weighted profile R-factor (R_wp_) improving from 21.9% to 21.5%. The plots in Fig. 5-c visually confirm this mathematical superiority; the difference between observed (I_obs_) and calculated (I_calc_) intensities is notably minimized in the asymmetric model compared to the symmetric counterpart.

**Table 4:** The values of the estimated values of peak positions, d-spacing, I_obs_, I_calc,_ and FWHM for the α-Alumina using the two fitting functions.

Pseudo Voigt							Pseudo Voigt “axial divergence asymmetry.”						
No	hkl	d(Å)	2θ	FWHM	I_obs_	I_calc_	No	hkl	D(Å)	2θ	FWHM	I_obs_	I_calc_
1	**012**	3.482685	25.556	0.00468	256.3	264.3	1	**012**	3.482549	25.557	0.00479	252	259
2	**104**	2.553042	35.121	0.00499	484.5	484.6	2	**104**	2.552914	35.123	0.00509	481.6	477.8
3	**011**	2.38119	37.748	0.00508	220.2	196.6	3	**011**	2.381119	37.749	0.00518	219	198.1
4	**006**	2.167148	41.64	0.00523	7.8	5.4	4	**006**	2.167012	41.643	0.00531	8.5	6.7
5	**113**	2.08698	43.319	0.0053	615.8	568	5	**113**	2.086902	43.321	0.00538	620.3	586
6	**202**	1.96566	46.142	0.00542	9.8	8.5	6	**202**	1.965596	46.143	0.00549	10.7	10.4
7	**024**	1.741342	52.508	0.00571	292	263.9	7	**024**	1.741274	52.51	0.00575	300	287.6
8	**116**	1.602745	57.449	0.00596	606.5	569	8	**116**	1.602669	57.452	0.00597	609.2	566.7
9	**211**	1.547771	59.692	0.00608	14.4	11.7	9	**211**	1.547725	59.694	0.00608	15	12.6
10	**122**	1.515889	61.08	0.00616	20.5	19.7	10	**122**	1.515842	61.082	0.00615	20.7	19.3
11	**214**	1.405595	66.461	0.00648	248.6	233.3	11	**214**	1.405545	66.464	0.00642	251.9	236.7
12	**300**	1.37478	68.152	0.00658	379.2	390.5	12	**300**	1.37474	68.154	0.00652	381.4	383.4
13	**125**	1.337045	70.354	0.00673	9.1	6.3	13	**125**	1.336994	70.357	0.00664	7	3.7
14	**208**	1.276521	74.231	0.00699	12.5	11.3	14	**208**	1.276457	74.235	0.00687	16.2	16.7
15	**10 10**	1.240117	76.798	0.00718	109.6	112	15	**10 10**	1.240043	76.804	0.00703	114.9	116.6
16	**217**	1.194095	80.343	0.00745	4.2	4.1	16	**217**	1.194043	80.347	0.00727	6.5	6.6
17	**223**	1.148068	84.279	0.00778	40.9	46	17	**223**	1.148032	84.282	0.00755	39.1	41
18	**128**	1.125054	86.418	0.00797	23.9	23.5	18	**128**	1.125003	86.423	0.00771	25.4	24.5
19	**02 10**	1.099894	88.906	0.00819	56.2	60	19	**02 10**	1.099835	88.912	0.00791	53.9	51.9

Furthermore, the FWHM analysis shows that the asymmetric model effectively captures the intensity “tails” at low scattering angles 2θ, where axial divergence is most pronounced, thereby preventing intensity from being erroneously treated as background noise. The refinement using the asymmetric model also facilitated a more physically realistic Electron Density Mapping (EDM). By accurately modelling the anisotropic thermal factors and suppressing Fourier artifacts (noise ripples) in the interstitial regions, the EDM generated from the asymmetric fit exhibited sharper and more localized electron density maxima. Although the theoretical density exhibited a shift from 4.115 g/cm^3^ to 3.985 g/cm^3^, the stability of the refined lattice parameters a = 4.762 Å, c = 13.002Å and unit cell volume across both models confirms the high phase stability of the α-Alumina produced via the modified Pechini method. Consequently, the inclusion of axial divergence asymmetry provides a truer representation of the nanoparticle crystal structure by correcting for instrumental smearing and optimizing intensity distribution across the diffraction pattern.

To accurately extract the intrinsic microstructural properties of the synthesized α-Alumina nanoparticles, such as effective crystallite size and microstrain, it is imperative to deconvolute the instrumental contribution from the total observed peak broadening. In the Bragg–Brentano geometry, the recorded Full Width at Half Maximum is a convolution of the instrumental resolution function (IRF) and the physical broadening arising from the specimen’s lattice dimensions and defects^55^. In this study, a high-purity standard corundum sample was utilized as an external reference to determine the instrumental parameters across the measured 2θ range. By modelling the standard’s profile with the previously validated pseudo-Voigt axial divergence asymmetry function, the Caglioti coefficients (U, V, W) were refined to define the instrumental baseline. This correction ensures that the subsequent calculations of grain size, typically via the size strain plot analysis, reflect the authentic nanostructural characteristics of the Pechini-derived alumina rather than artifacts of the diffractometer’s optical path. The following equations are used in the correction of the FWHM values.

5 $${\mathrm{FWHM}}_{{({\mathrm{corrected}})}} = \, \left( {{\mathrm{FWHM}}_{{{\mathrm{Refined}}}} {-}{\text{ FWHM}}_{{{\mathrm{diffractometer}}}} } \right)$$

6 $${\mathrm{FWHM}}_{{({\mathrm{corrected}})}} = \, \left( {{\mathrm{FWHM}}^{{2}}_{{{\mathrm{refined}}}} {-}{\text{ FWHM}}^{{2}}_{{{\mathrm{diffractometer}}}}}\right)^{{1}/{2}}$$

**Fig. 6:**
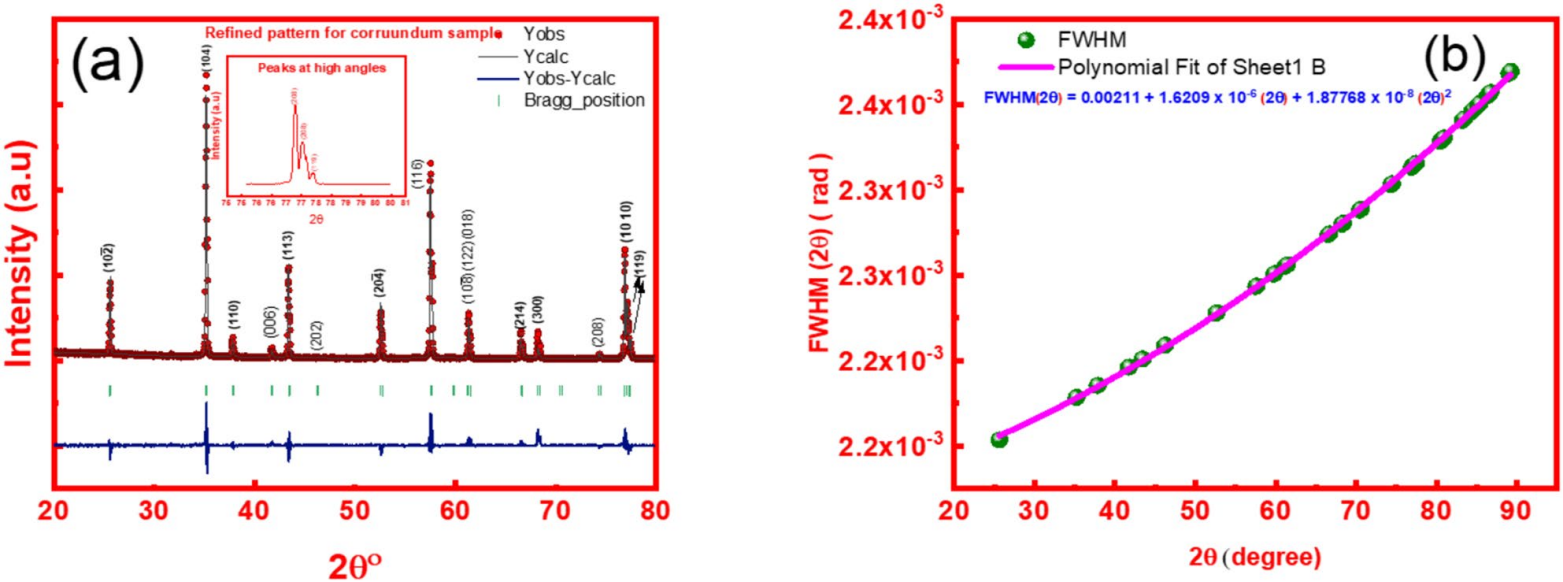
(**a**) The refinement for the reference sample; (**b**) The resolution profile.

The linear model (Eq. 1) is physically valid when the peak profiles are purely Lorentzian, a condition typically associated with size-broadened nanocrystals with significant “tail” intensity. Conversely, the squared model (Eq. 2) assumes a purely Gaussian distribution, which accurately describes broadening dominated by instrumental resolution and lattice strain in a Bragg–Brentano setup. Given that the Rietveld refinement of the synthesized α-Alumina yielded mixing parameters (η) effectively approaching zero (0.000061 and 0.023062) in both the symmetric and asymmetric models, the diffraction profiles are demonstrated to be predominantly Gaussian in character. Therefore, for the present work, the squared relationship is recommended as the most scientifically rigorous approach for instrumental correction. Utilizing this method ensures that the deconvolution process aligns with the refined pseudo-Voigt parameters, providing a precise baseline for the subsequent determination of crystallite size and lattice strain. The value of FWHM_diffractometer_ can be estimated using the standard sample as mentioned earlier, the way for this is performing XRD Diffractometry for the reference samples under the same conditions for the tested samples. Performing the Rietveld refinement for it, after that, the FWHM of the reference sample is extracted for the whole range, then its values are subtracted from the FWHM of the tested samples according to Eq. 5. Figure 6 illustrates the refinement for the reference sample and the resolution profile Table 5.

**Table 5:** The corrected values for the FWHM for the two fitting functions.

Pseudo Voigt		Pseudo Voigt “axial divergence asymmetry.”	
Peak position	FWHM_Corrected_	Peak position	FWHM_Corrected_
25.556	0.004166137803	25.557	0.004289150229
35.121	0.004505535107	35.123	0.004614427519
37.748	0.004609882284	37.749	0.004711672515
41.64	0.004773569249	41.643	0.004862529245
43.319	0.004847578576	43.321	0.004930045914
46.142	0.004976749563	46.143	0.005047244911
52.508	0.005290833536	52.51	0.005329058036
57.449	0.005557783901	57.452	0.005565951648
59.692	0.005686113039	59.694	0.005679122626
61.08	0.005767874111	61.082	0.005751061748
66.461	0.006102973866	66.464	0.006044713327
68.152	0.006214543129	68.154	0.006142076095
70.354	0.006364682024	70.357	0.006272929562
74.231	0.006643001967	74.235	0.006514862169
76.798	0.006837957994	76.804	0.006683945688
80.343	0.007122194308	80.347	0.006929804156
84.279	0.007460403207	84.282	0.007221792851
86.418	0.007655098037	86.423	0.007389802281
88.906	0.007892059395	88.912	0.007594040862

**Fig. 7:**
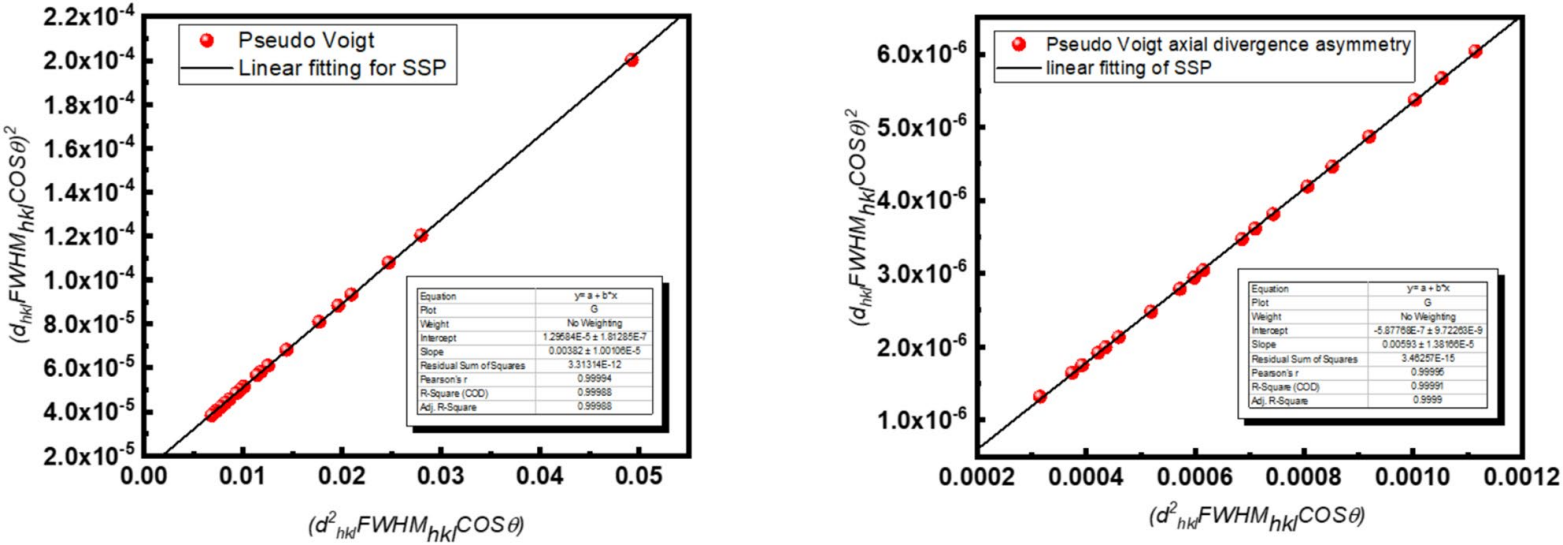
The results obtained from the SSP application for the two fitting functions.

To characterize the microstructural evolution of the synthesized α-Alumina nanoparticles, a comprehensive evaluation of crystallite size and lattice strain was performed using the Size-Strain Plot (SSP) method^18,56^. Unlike the standard Scherrer equation, which attributes peak broadening solely to size effects, or the Williamson-Hall (W–H) plot, which often assumes a uniform distribution of strain^56,57^, the SSP method offers a more physically robust deconvolution of these two components by utilizing a weighted profile approach. In this model, the total broadening (FWHM_hkl_) is treated as a convolution, with size-related broadening following a Lorentzian distribution and strain-induced broadening following a Gaussian distribution. This distinction aligns with the refined pseudo-Voigt parameters previously established for this corundum phase. The following equation states the size strain plot:

7 $$\left(dhkl FWHMhkl Cos\theta \right)2=\frac{K\lambda }{D} \left({d}_{hkl}^{2} {FWHM}_{hkl} Cos\theta \right)+{(\frac{\varepsilon }{2})}^{2}$$

where *d*_*hkl*_ is the interplanar spacing in angstroms, *FWHM*_*hkl*_ is the instrumental-corrected FWHM (in radians), K is the shape factor (typically 0.94), **λ** is the X-ray wavelength, typically 1.5406 Å for Cu-Kα, D is the apparent crystallite size, and ε is the apparent lattice strain. By plotting *(d*_*hkl*_* FWHM*_*hkl*_* Cosθ)*^*2*^ against $\left({d}_{hkl}^{2} {FWHM}_{hkl} Cos\theta \right)$ The apparent crystallite size D and the intrinsic lattice strain ε can be extracted from the slope and Y-intercept, respectively. This method is particularly advantageous for the Pechini-derived α-Alumina calcined at 1100°, as it accounts for the potential strain developed during the high-temperature phase transformation to the corundum structure while providing a truer estimation of the effective grain dimensions. Figure 7 illustrates the results obtained from the SSP application for the two fitting functions.

**Fig. 8:**
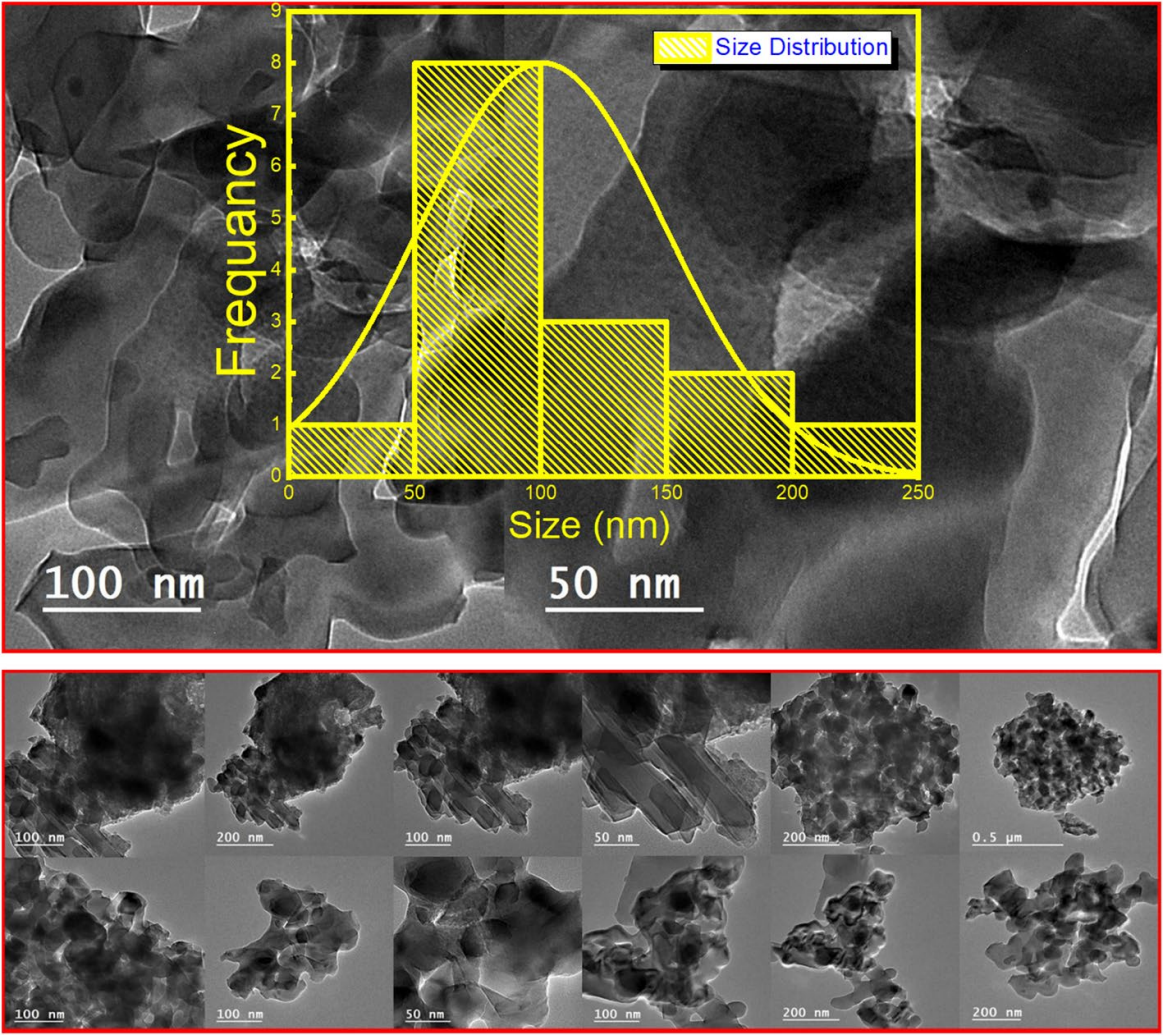
The HR-TEM images.

From Fig. 7, the size strain plot for the pseudo-Voigt and the pseudo-Voigt axial divergence asymmetry are investigated; on the other hand, HR-TEM analysis via the image mode has been investigated to estimate the average crystallite size as a comparison between the calculated and the actual size for the α-Alumina sample. Figure 8 illustrates the HR-TEM images, and the Table 6 summarizes the results obtained from it and the fitting functions.

**Table 6:** The results obtained from it and the fitting functions.

Pseudo Voigt	Pseudo Voigt “axial divergence asymmetry.”
Crystallite size = 40 ± 1.54 nm	Crystallite size = 24.4 ± 1.2 nm
Strain = 0.0071994442 ± 0.0002	Strain = 0.00153323188 ± 0.0002
HR-TEM results obtained from the histogram	
Crystallite size is about 100 ± 1.56 nm nm	

The comprehensive microstructural analysis of the synthesized α-Alumina nanoparticles reveals a significant correlation between the refined mathematical models and the physical reality of the corundum phase. The transition from a standard symmetric Pseudo-Voigt function to one incorporating axial divergence asymmetry proved essential for capturing the true peak profiles, as evidenced by the reduction in the weighted profile R-factor (R_wp_) from 21.9% to 21.5% and the improvement in the goodness of fit χ^2^ = 2.48. This mathematical refinement directly influenced the Size-Strain Plot (SSP) results, where the asymmetric model yielded a crystallite size (D) of 24.4 nm and a remarkably low lattice strain of 0.0015, compared to the 40 nm and 0.0072 obtained from the symmetric model. A critical observation in the SSP for the asymmetric model was the emergence of a slightly negative Y-intercept.

In the framework of the SSP method, where the intercept is defined as $\frac{{\varepsilon }^{2}}{2}$ A negative value is mathematically non-physical and serves as a diagnostic indicator of negligible intrinsic lattice strain. This suggests that the high-temperature calcination at 1100 °C provided sufficient thermal energy to facilitate a complete phase transformation to the thermodynamic α-Alumina phase while effectively relieving internal stresses and defects. Furthermore, the negative intercept implies that the axial divergence correction successfully removed the instrumental “smearing” that often artificially inflates strain values in standard models. Consequently, the broadening of the diffraction peaks in this sample is almost entirely size-dependent rather than strain-dependent.

The direct physical evidence provided by HR-TEM imaging further elucidates the nature of the powder, showing irregular, plate-like nanoparticles with a mean size of approximately 100 nm according to the histogram analysis. The discrepancy between the XRD-derived coherent scattering domain (24.4 nm) and the TEM-observed particle size (100 nm) suggests that the particles are polycrystalline aggregates formed during the Pechini polyesterification and subsequent high-temperature sintering.

Ultimately, the integration of Rietveld refinement, SSP analysis, and HR-TEM confirms that the modified Pechini method produces high-purity, stress-free α-Alumina nanoparticles characterized by small crystallographic domains within larger aggregated morphological units. The observed difference between the 24.4 nm crystallite size calculated from the axial divergence asymmetry SSP model and the approximately 100 nm particle size derived from the HR-TEM histogram is primarily due to the distinction between a "coherent scattering domain" and a “physical particle”. XRD is a volume-averaging technique that measures the size of coherent domains where the periodic lattice arrangement is uninterrupted by significant defects or grain boundaries. Conversely, HR-TEM imaging provides a direct visualization of the physical boundaries of a particle, which often consists of multiple small crystallites or "sub-grains" aggregated together.

## FTIR analysis

**Fig. 9:**
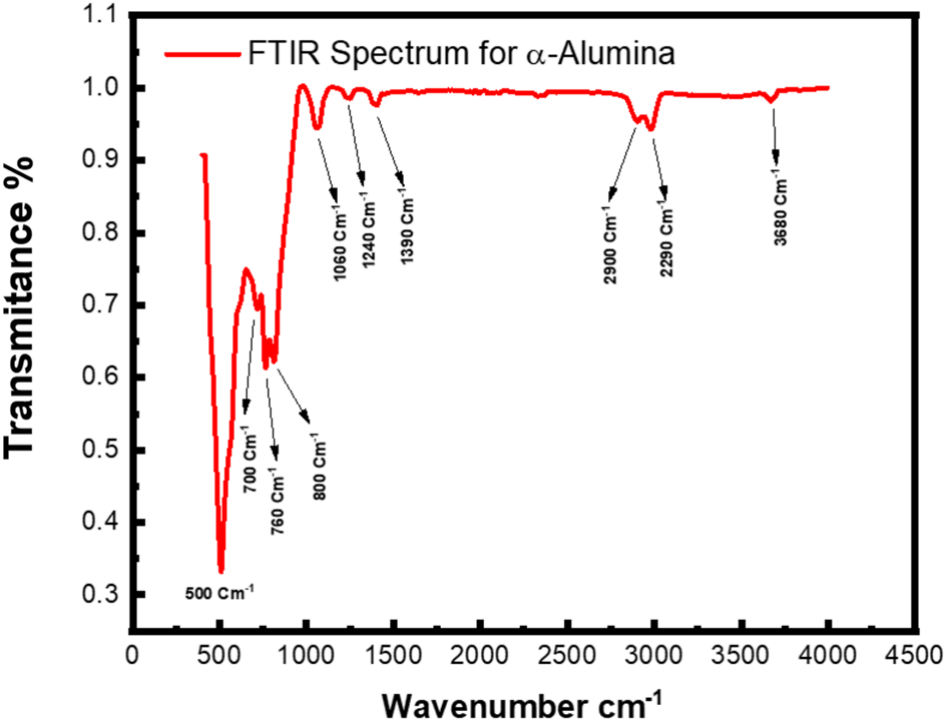
The FTIR spectrum for the α-Alumina.

To complement the structural and microstructural analysis, Fourier Transform Infrared (FTIR) spectroscopy was performed in Attenuated Total Reflectance (ATR) mode over the spectral range of 400 to 4000 cm^-1^. This technique serves as a sensitive probe for the local vibrational modes and the chemical bonding environment, offering a molecular-level perspective that complements X-ray diffraction data. By utilizing the ATR mode, the surface and bulk characteristics of the synthesized α-Alumina nanoparticles can be examined with high precision, specifically focusing on the characteristic Al-O stretching and bending vibrations in the fingerprint region. Figure 9 illustrates the transmittance spectrum for the tested sample.

From the figure above, the band around 500 cm^-1^ is attributed to the aluminium oxide stretching modes, especially AlO_6_ octahedra^58–60^. In contrast, the band around 700 cm^-1^ may be attributed to the condensed Al–O–Al stretching and bending modes. Specifically, the peaks at 760 cm^-1^ and 800 cm^-1^ are often associated with Al–O–Al lattice vibrations (phonon modes) of the AlO_6_ framework^61^. The very low intensity of bands between 1000 and 1500 cm^-1^ indicates nearly complete decomposition of the Pechini precursors (citric acid and PEG 400):

• 1060 cm^-1^ and 1240 cm^-1^, which are associated with the bending modes of surface hydroxyl groups (Al–OH) and potentially Al-O-C stretching vibrations from trace surface-active species^62,63^.
• 1390 cm^-1^: These species typically form via the chemisorption of atmospheric CO_2_ onto the active basic sites (Lewis base centers) of the alumina surface upon exposure to the ambient environment. Given the high calcination temperature of 1100°C, which significantly exceeds the thermal decomposition threshold of the organic precursors used in the synthesis, these features are interpreted as surface-limited phenomena rather than bulk impurities. The persistence of these surface groups is a characteristic trait of high-surface-area metal oxides, where the coordination-unsaturated surface ions remain chemically active even after high-temperature processing. This interpretation is consistent with the ‘high-purity’ nature of the crystalline core, as evidenced by the sharp Bragg reflections in the XRD patterns and the absence of any secondary amorphous organic phases^64,65^.
• 2900 cm^-1^: This small band is assigned to the symmetric and asymmetric C–H stretching modes of residual organic chains^66^.

The high calcination temperature, 1100 °C typically removes bulk moisture, but surface effects remain visible:

- 2290 cm^-1^: Attributed to the asymmetric stretching of atmospheric CO_2_ molecules adsorbed onto the surface of the nanoparticles^67^.
- 3680 cm^-1^: This sharp, high-frequency band corresponds to the stretching vibrations of isolated surface hydroxyl (O–H) groups. Its sharpness suggests these are free hydroxyls on the nanoparticle surface rather than hydrogen-bonded bulk water^68^.

## Optical study

**Fig. 10:**
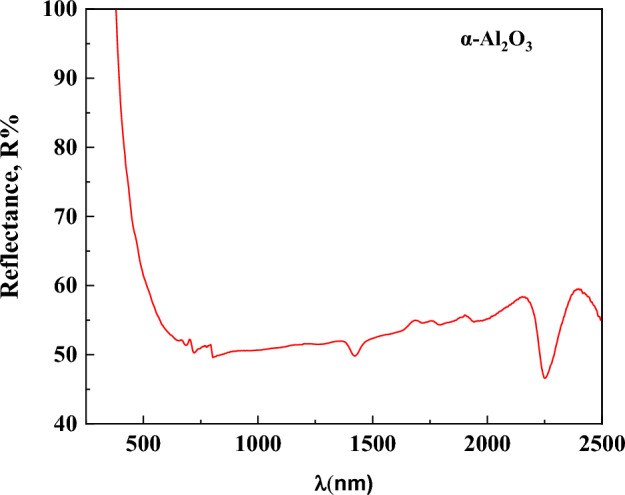
Reflectance spectra of α-Al_2_O_3_ as a function of wavelength.

The wavelength-dependent reflectance spectrum of α-Al_2_O_3_ is shown in Fig. 10. In the UV region below about 300 nm, the curve shows a marked increase in reflectance. As shown in Fig. 10, reflectance stability in the visible range (400-800nm). In the infrared region (800-2500nm), there are many dips in the spectrum.

**Fig. 11:**
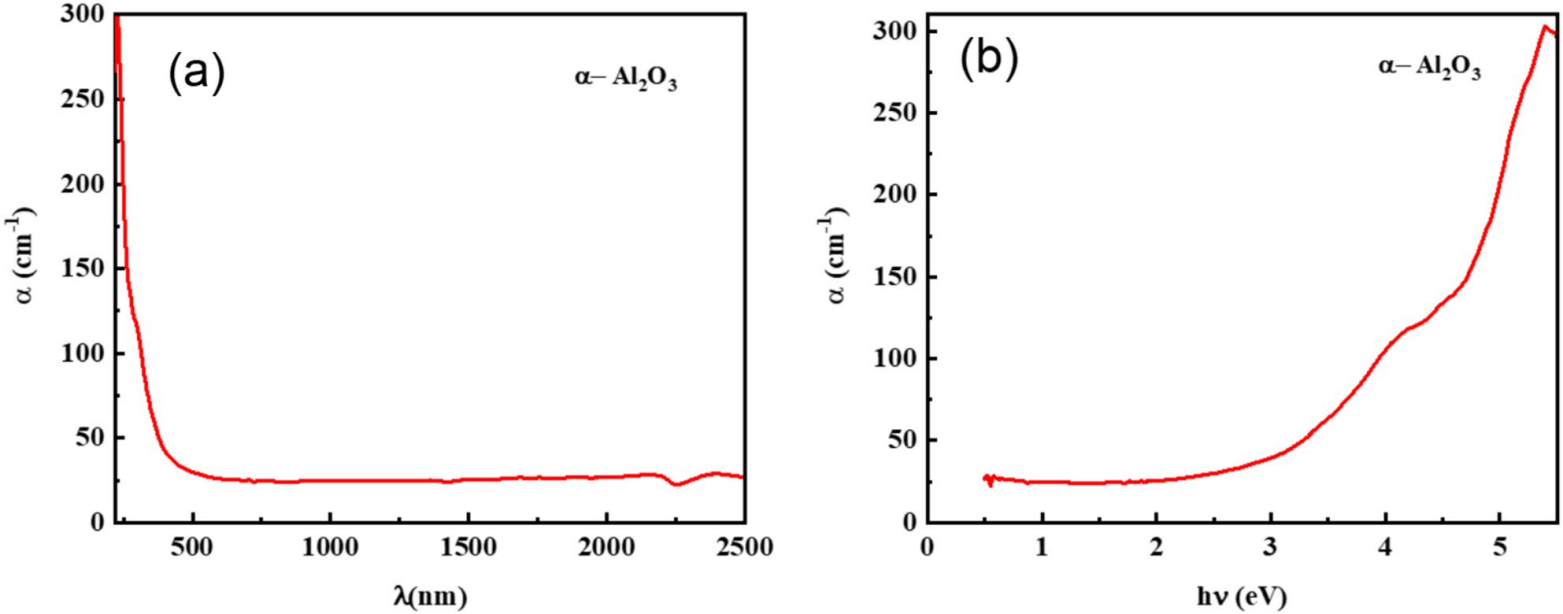
(**a**) Absorption coefficient as a function of wavelength. (**b**) Absorption coefficient as a function of photon energy for α-Al_2_O_3_.

**Fig. 12:**
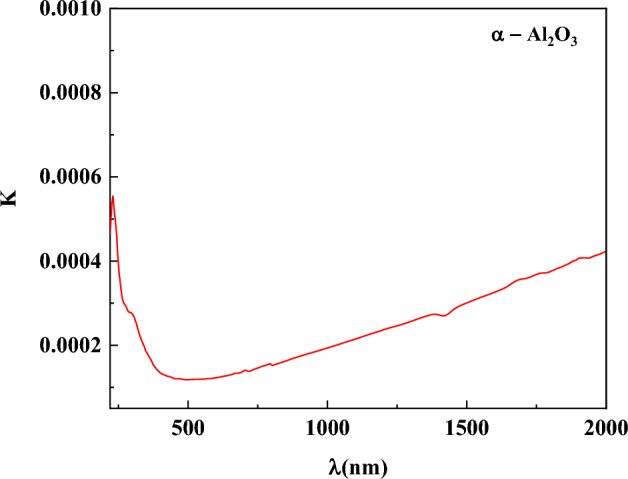
Spectra of extinction coefficients k as a function of wave length.

The extinction coefficient, k, is a property that describes how strongly a film absorbs or reflects light at a specific wavelength. It can be computed using this formula:

8 $$k=\frac{\alpha \lambda }{4\pi }$$

where α is the absorption coefficient, it can be found using the following formula:

9 $$F\left(R\right)=\frac{k}{S}=\frac{{\left(1-R\right)}^{2}}{2R}$$

where k is The Absorption molar coefficient (units: cm^-1^ or m^-1^). This represents how much light is absorbed by the material at a specific wavelength. The scattering coefficient is denoted by S, the Kubelka–Munk function by F(R), and the reflectance by R. The spectrum behavior of the absorption coefficient for α-Al_2_O_3_ is shown in Fig. 11. Figure 11(a) illustrates that α-Al_2_O_3_ has a significant absorption coefficient in the ultraviolet spectrum. The absorption coefficient reduces in the visible range and is extremely low value in the near-infrared spectrum. This makes the material perfect for use as a driving mirror and window screen coating to block the impact of bright light on drivers’ eyes as they are approaching cars^69–72^. (Fig. 11(b)) illustrates that the absorption coefficient increases with increasing photon energy, which may be due to fundamental band gaps predominating^73^. As seen in Fig. 11(a) and Fig. 12, the behavior of the spectra of k and α as a function of wavelength is comparable. At low wavelengths, the reduction of k is noticeable and appears to be constant across the majority of the wavelength region. This makes sense since the photons are first absorbed or reflected at low wavelengths, and then they are transmitted via high wavelengths. However, a drop in α signifies a decrease in the optical energy gap^74,75^.

The following formula is used to get the average refractive index, n, in the wavelength range of 500–2500 nm.

10 $$n=\frac{1+{R}^{0.5}}{1-{R}^{0.5}}$$

**Fig. 13:**
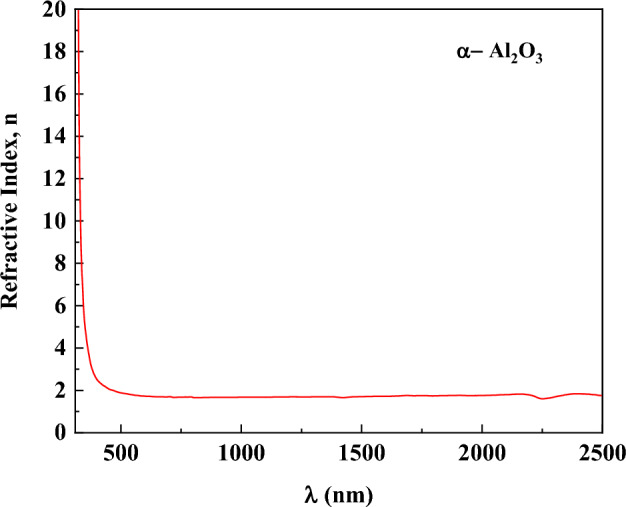
Refractive indices of α-Al_2_O_3_ as a function of wavelength.

Using Eq. (9), the refractive index n(λ) is calculated and shown in Fig. 13. The refractive index’s behavior indicated normal dispersion^76,77^

For powder materials, the optical energy gap E_g_ is estimated using the Kubelka–Munk method:

11 $$\left(F\left(R\right)h\upsilon \right)=A{\left(h-{E}_{g}\right)}^{n}$$

**Fig. 14:**
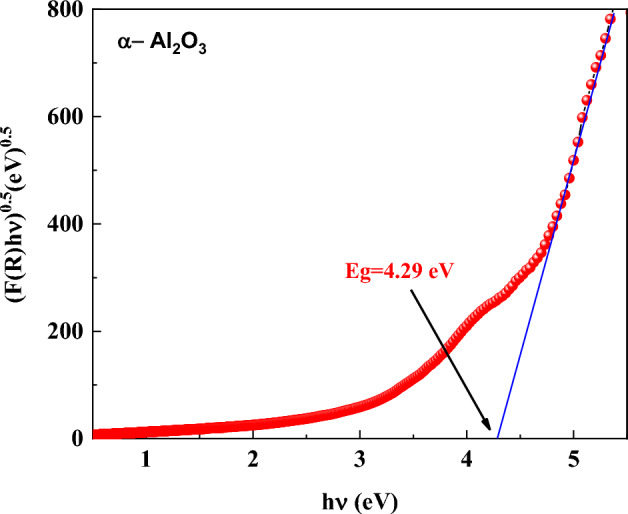
Relation between (F(R)hν)^0.5^ and the photon energy.

Figure 14 shows the photon energy as a function of the absorption coefficient represents (F(R)hν)^0.5^. The optical band gap, E_g_ of α-Al_2_O₃, is obtained by extrapolating the linear section of the curve to the energy axis. Based on the crossing point, the band gap is calculated to be 4.29 eV.

The definition of the complex dielectric function ε(ω) is:

12 $$\varepsilon \left(\omega \right)={\varepsilon }{\prime}\left(\omega \right)+{\varepsilon }^{{\prime}{\prime}}(\omega )$$

where the real and imaginary components of are denoted by ε′ and ε″. Figure 6 shows the spectra of both ε′ and ε″. The expression for ε′ is as follows:

13 $${\varepsilon }{\prime}={n}^{2}-{k}^{2}={\varepsilon}_{L}-\frac{{e}^{2}}{4{\pi }^{2}{\varepsilon}_{o}}\left(\frac{{N}_{c}}{{m}^{*}}\right){\lambda }^{2}$$

where m* is a charge carrier’s effective mass, ε_o_ is the free space permittivity (8.854 × 10^–12^ F/m), e is the electron charge, N_c_ is the concentration of free carriers, and ε_L_ is the lattice dielectric constant. Figure 6 shows the intercept and slope of the plot ε′ vs. λ^2^ from which the values of ε_L_ and ratio N_c_/m* can be derived. The following formula can be used to get the plasma frequency (ω_P_)^78^:

14 $${\omega}_{P}^{2}=\frac{N}{{m}^{*}}\left(\frac{{e}^{2}}{{\varepsilon}_{o}{\varepsilon}_{L}}\right)$$

**Fig. 15:**
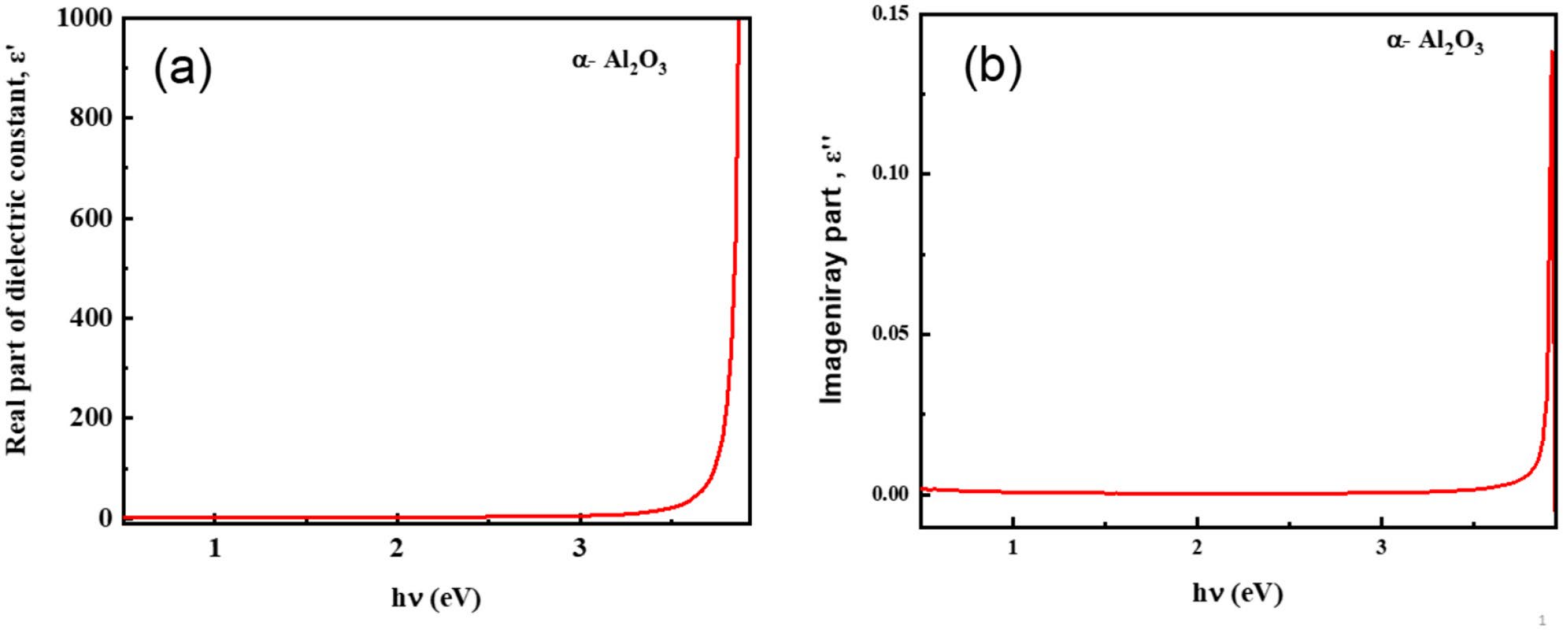
α-Al_2_O_3_ Spectra of (**a**) the real components of the complex dielectric constant ɛʹ, and (**b**) the imaginary components of the complex dielectric constant ɛʹʹ.

**Fig. 16:**
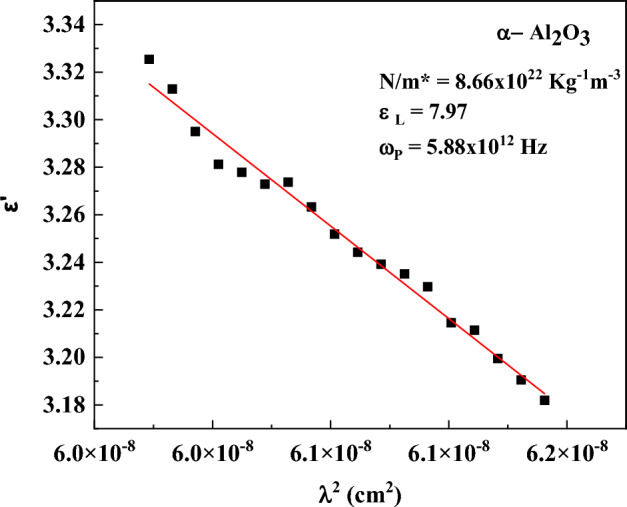
Optical parameters for α-Al_2_O_3_; free carrier concentration per effective mass, N/m*, the lattice dielectric constant ε_L_, and the electron plasma frequency, ω_P_.

The calculation of the optical parameters ɛ_L_, N/m* and ω_P_ is shown in Fig. 15, 16.

The inelastic scattering process that takes place during charge transfer and charge conduction mechanisms in thin-film materials is usually the cause of power loss in the form of heat; this is known as the loss factor tan δ, which indicates the rate at which mechanical power is lost. The dissipation factor (tan δ) is another name for the power loss caused by a dipole oscillation in a dissipative medium. The relationship is used to estimate the loss factor, or the ratio of ε_1_ to ε_2_^79^. The loss factor is calculated by:

15 $$tan\delta =\frac{{\varepsilon }^{{\prime}{\prime}}}{{\varepsilon }{\prime}}$$

**Fig. 17:**
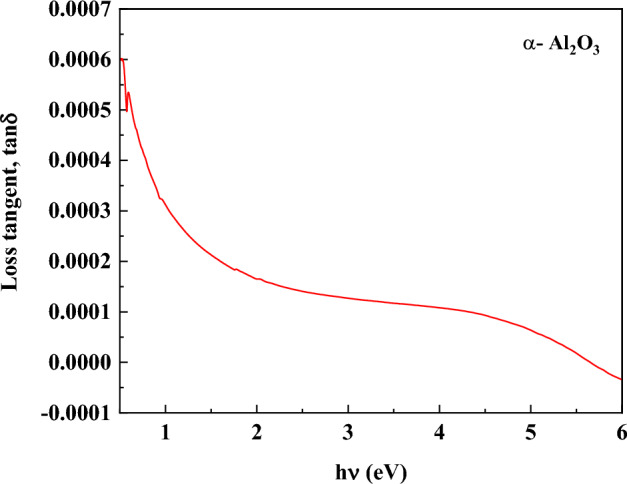
Variation of the dielectric loss tangent (tan δ) of α-Al_2_O_3_ as a function of photon energy.

The loss tangent measures the power loss rate in an oscillating dissipative system. Figure 17 illustrates the loss factor of α-Al_2_O_3_ as a function of the incident photon energy. Clearly, the variance loss curve behaves in the opposite way from ε’’. Since ε’’ is smaller than ε’, α-Al_2_O_3_ loses less energy overall. Due to their lower energy losses and incoming radiation scattering, α- Al_2_O_3_ considered to have better optical quality^80,81^.

Figure 17 presents the variation of the dielectric loss tangent (tan δ) of α- Al_2_O_3_ as a function of photon energy in the range of 0.5–6.0 eV. The loss tangent, defined as the ratio of the imaginary to the real part of the complex dielectric function (tan δ = ε″/ε′), provides a direct measure of the energy dissipation within the material during interaction with electromagnetic radiation.

At low photon energies (hυ < 1 eV), tan δ exhibits relatively higher values, which can be attributed to dielectric relaxation processes associated with defect dipoles, localized states, and lattice imperfections. In this energy regime, polarization mechanisms can track the oscillating electric field, leading to increased energy dissipation. Similar behavior has been reported for high-purity alumina and sapphire, where residual defects and impurity-related relaxation dominate the dielectric loss at low frequencies and energies^78^.

As the photon energy increases, tan δ decreases rapidly and approaches a nearly constant, very low value in the intermediate energy range (≈1–4 eV). This behavior indicates the suppression of slow relaxation mechanisms and the dominance of electronic polarization, which responds more efficiently to high-frequency optical fields with minimal energy loss. The weak variation of tan δ in this region reflects the intrinsic insulating nature of α- Al_2_O_3_ and its low density of free charge carriers, consistent with previous optical and dielectric studies^82–84^.

At higher photon energies approaching the optical band gap of α- Al_2_O_3_ (≈4–6 eV), the loss tangent further decreases and tends toward minimal values. This trend is associated with the absence of significant mid-gap states and the limited contribution of inter-band transitions to dielectric loss below and near the absorption edge. The extremely small tan δ values observed in this region confirm that α- Al_2_O_3_ dissipates negligible electromagnetic energy, highlighting its exceptional optical quality and dielectric stability^85–90^.

**Fig. 18:**
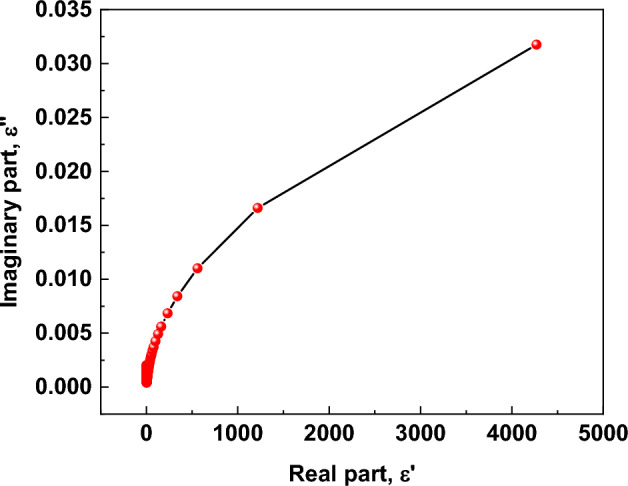
Correlation between the imaginary (ε″) and real (ε′) parts of the complex dielectric function of α-Al_2_O_3_.

Overall, the consistently low loss tangent across the investigated photon energy range demonstrates that α- Al_2_O_3_ is an excellent low-loss dielectric material. These characteristics make it highly suitable for advanced optical coatings, high-frequency photonic devices, ultraviolet optoelectronics, and applications requiring minimal energy dissipation under electromagnetic excitation (Figure 18).

Figure 9 presents the correlation between the imaginary part, ε″, and the real part, ε′, of the complex dielectric function of α-Al_2_O_3_. This ε″–ε′ representation provides a comprehensive picture of how energy dissipation and polarization evolve in response to an electromagnetic field and links directly to fundamental optical parameters, including the loss tangent (tan δ), the extinction coefficient k(λ), and the optical band gap E_g_.

In dielectric materials, the complex dielectric function ″ϵ = ϵ′ + iϵ″ embodies the entire optical response: ε′ quantifies the ability of bound charges to polarize and store energy, while ε″ represents all energy dissipation mechanisms, including electronic transitions and phonon or defect-induced losses^86,87^. The dielectric loss tangent, defined as tanδ = ϵ″/ϵ′, therefore directly reflects the relative contribution of dissipative processes to the overall dielectric response.

At low ε′ values, ε″ increases sharply relative to ε′. This regime corresponds to photon energies below or near the onset of significant inter-band absorption, where dielectric relaxation due to defects, localized vibrational modes, and low-energy charge fluctuations dominates. These processes contribute disproportionately to ε″, leading to higher tan δ^78,88^. Simultaneously, the extinction coefficient k(λ), related to the absorption coefficient by k = αλ/4πk, also exhibits elevated values at short wavelengths, indicating increased optical absorption from defect and phonon-assisted transitions. The concurrence of high ε″ and k(λ) in this region underscores the role of extrinsic loss mechanisms that are not directly related to intrinsic electronic structure.

As ε′ increases, corresponding to higher photon energies approaching the visible and near-UV regions, ε″ continues to rise but with a reduced slope, resulting in a marked decline in tan δ. This trend highlights the transition from relaxation-dominated losses to polarization dominated by bound electron displacement. In this intermediate region, intrinsic electronic polarization contributes strongly to ε′ while contributing comparatively little to ε″, leading to suppressed dielectric loss. The extinction coefficient k(λ) in this spectral range also decreases and tends toward constant values, reflecting minimal absorption and confirming the dominance of transparent, low-loss electronic response. This behavior is consistent with the known optical properties of wide-band-gap insulators, where electronic transitions are virtual and do not significantly dissipate energy until photon energies approach the band-edge region^91,92^.

At high ε′ values, ε″ scales nearly linearly with ε′ but remains minimal in magnitude, which corresponds to photon energies closer to the optical band gap, E_g_. For α- Al_2_O_3_, Kubelka–Munk analysis yields an E_g_ of approximately 4.29 eV, characteristic of a wide-band-gap dielectric^76,93^. In this high-energy regime, real inter-band transitions begin to contribute, but the scarcity of mid-gap states and the broad gap suppress substantial absorption below E_g_. Consequently, both ε″ and k(λ) remain low, and tan δ approaches negligible values. Such low-loss behavior is a hallmark of high-quality dielectric materials and is corroborated by recent precision measurements of dielectric loss in wide-band-gap oxides showing tan δ values approaching 10^–6^ or lower outside resonance regions^94^.

The ε″–ε′ correlation thus encapsulates the full dielectric behavior of α-Al_2_O_3_: an initial dominance of relaxation and defect-induced losses at low polarization, a transition to intrinsic electronic polarization with declining relative loss, and, near the optical band edge, minimal ε″ despite increasing ε′, reflecting the wide band gap and inherent transparency of the material. Linking this behavior to tan δ and k(λ) not only confirms the material’s low-loss optical performance but also highlights the critical role of E_g_ in defining the onset of absorption and dissipation mechanisms. Collectively, these results underscore the suitability of α- Al_2_O_3_ for applications that demand minimal dielectric loss, high transparency, and stable optical response, including low-loss optical coatings, photonic devices, and high-frequency dielectric components (Table 7).

**Table 7:** Quantitative comparison with literature.

Parameter	This work	Literature / Reference Values	Comments
Optical band gap, E_g_	** ~ **4.29 eV ± 0.05 EV	Optical constants data generally show band gap onset near ~ 4.0–5.5 eV for Al₂O₃; Palik & refractiveindex.info compilations show absorption edge in the deep UV (≈6–8 eV) for α-Al₂O₃ single crystals^95^	Values vary with sample, measurement, and definition (onset vs. Tauc); E_g_ is within the expected range for indirect transitions in porous/film forms
Refractive index (n)	~ 1.6–2.0 (± 1.2) (visible–NIR)	Sapphire n ~ 1.75 at 1.06 µm and decreases with wavelength^96^	n is slightly lower; it fits the trend of dispersion and indicates high transparency and low density of defect states
Extinction coefficient (k)	~ 10^–4^ – 10^–3^	Literature reports very low k for sapphire in visible–NIR (often < 10⁻^3^)^96^	Low k aligns with reported transparent behavior up to ~ 5 µm
Real dielectric constant (ε′)	Up to ~ 3.3 × 10^3^ *(spectral)*	Optical ε′ varies strongly with energy, > 10^2^ near UV^97^	Optical ε′ can be orders of magnitude higher than published due to high polarizability at UV energies
Imaginary dielectric constant (ε″)	≤ ~ 0.03	Optical ε″ for sapphire is typically very small in transparent regions^95^	Small ε″ is expected in the visible/NIR transparency window
Loss tangent (tan δ)	~ 10^–4^–10^–6^	Commercial sapphire loss tangent ~ 10⁻^4^ at microwave frequencies; even lower optically ^98^	Optical tan δ values are consistent with ultra-low optical losses
Lattice dielectric constant (ε_L_)	7.97 ± 0.15	Static dielectric constant ~ 9–11.5 ^99^	Close to the expected range considering different measurement regimes
Free carrier N/m*	8.66 × 10^22^ kg⁻^1^·m^-3^	Sapphire is an insulator with negligible free carriers^100^	Relative magnitude is consistent with insulating behavior
Plasma frequency (ωₚ)	5.88 × 10^12^ Hz	Not commonly reported for sapphire optical spectra, but expected to be low for insulators	Consistent with a lack of free carrier response

The set of optical parameters extracted in this work is consistent both internally and with established literature trends for α-Al_2_O_3_. The refractive index spectrum, exhibiting values in the range ≈1.6–2.0 across the visible-near-infrared region, aligns with referenced optical constants for sapphire, which report n ≈ 1.75 at 1.06 µm and a monotonic decrease with increasing wavelength within the transparency window. The extinction coefficient k(λ), on the order of 10⁻4–10⁻3, and the very small imaginary dielectric constant ε″ (≤ 0.03) are consistent with literature values for low-loss sapphire within its transparent spectral range up to ~ 5 µm. The extracted optical band gap (E_g_ ≈ 4.29 eV) falls in the range commonly reported when applying Tauc or indirect transition models to experimental absorbance data; although the fundamental electronic band edge of α-Al_2_O_3_ is known to occur at higher energies (≈ 6–8 eV) in deep UV measurements, indirect optical models often yield lower effective values due to phonon or defect contributions^95^. The high values of ε′ at optical photon energies, coupled with the low tan δ values (~ 10^–4^ -10^–6^), reflect strong electronic polarization with minimal dissipation, and are thus mutually supportive: the low ε″ and tan δ agree with low k(λ) and the absence of free carrier absorption typical of wide-band-gap insulators. Finally, the lattice dielectric constant (ε_L_ ≈ 7.97) and negligible free carrier concentration are physically consistent with the static dielectric measurements reported for α-Al_2_O_3_, which fall in the range of ≈ 9–11 at MHz frequencies.

## Conclusion

In this study, high-purity α-Alumina nanoparticles were successfully synthesized via a modified Pechini sol–gel route, followed by a rigorous investigation into their structural, microstructural, and optical properties. Rietveld refinement confirmed the formation of a phase-pure corundum lattice with R-3c symmetry. A key methodological contribution of this work is the implementation of axial divergence asymmetry profile fitting, which mathematically accounts for instrumental aberrations at low 2θ angles. Unlike standard symmetric models, this high-fidelity deconvolution isolated geometric broadening from intrinsic lattice effects, enabling a superior statistical fit and a more precise determination of Al–O bond lengths and angles. Microstructural analysis using the Size-Strain Plot (SSP) method, with FWHM values standardized in radians, yielded a volume-weighted crystallite size of 24.4 nm. The refinement yielded a physically consistent mixing parameter (η = 0.000061), indicating a predominantly Gaussian peak shape, while the SSP analysis revealed a stress-free crystalline lattice with negligible internal strain. These findings were corroborated by HR-TEM imaging, which showed spherical-like polycrystalline aggregates with an average diameter of 100 nm. Furthermore, FTIR spectroscopy confirmed the chemical integrity of the phase; while bulk vibrations were dominated by octahedral AlO_6_ modes The absence of residual organic precursors further verified the efficiency of the 1100 °C calcination process. Comprehensive optical characterization based on diffuse reflectance spectroscopy enabled the extraction of key optical constants and dielectric parameters within a unified framework. The material exhibits a wide optical band gap (E_g_ ≈ 4.29 eV), normal refractive index dispersion, and an extremely low extinction coefficient across the visible and near-infrared regions, confirming high optical transparency. Complex dielectric analysis revealed a strong real dielectric response accompanied by an exceptionally small imaginary component, resulting in ultra-low dielectric loss (tanδ ≈ 10^–4^—10^–6^). The consistency among ε’, ε’’, tanδ, together with the extracted lattice dielectric constant, demonstrates that the optical response is dominated by intrinsic low-loss electronic polarization. Overall, the strong correlation established between structural integrity and low-loss optical behavior provides new insights into energy storage and dissipation mechanisms in wide-band-gap oxides. These findings confirm that α-Al₂O₃ nanoparticles are highly promising candidates for ultraviolet optoelectronic devices, low-loss photonic components, and high-frequency dielectric applications. This refined methodology establishes a more reliable benchmark for structure–property relationships in phase-pure corundum, ensuring the high degree of structural accuracy required for precision technological applications.

## Data Availability

The data that support the findings of this study are available upon reasonable request from the authors.
